# The complete chloroplast genome sequence of *Pennisetum flaccidum* (Poaceae)

**DOI:** 10.1080/23802359.2021.1920497

**Published:** 2021-08-04

**Authors:** Wangsuo Liu, Yingzhong Xie, Kaiyang Qiu, Pengbo Zhao, Haiquan Li, Xueqin Hu, Hu Miao

**Affiliations:** a School of Agriculture, Ningxia University, Yinchuan, PR China; b Ningxia Research Center of Engineering and Technology for Prataculture and Animal Husbandry, Yinchuan, PR China; c Ningxia Grape Wine and Desertification Prevention Technical College, Yinchuan, PR China

**Keywords:** Poaceae, *Pennisetum flaccidum*, chloroplast genome, phylogenetic tree

## Abstract

*Pennisetum flaccidum* Grisebach is a typical high-quality forage and adrought-tolerant grass. In this study, we firstly reported the complete chloroplast (cp) genome of *P. flaccidum*, which was 138,336 bp in length, including a pair of inverted repeats (IR: 22,293 bp), a large single copy (LSC: 81,329 bp), and a small single copy (SSC: 12,421 bp) region. A total of 131 genes were annotated, containing seven *rRNA* genes, 38 *tRNA* genes, and 86 protein-coding genes. The GC content of the cp genome was 38.63%. The maximum-likelihood (ML) phylogenetic tree indicated that *P. flaccidum* was closely related to *P. cetaceum* in Poaceae.

*Pennisetum flaccidum* Grisebach is a plant species of the genus of *Pennisetum* in the family of Poaceae and widely distributes in northern China with high quality and excellent palatability (Joshi and Ludri 1967; Yu et al. 2009). *P. flaccidum* has strong drought tolerance and can reproduce and grow multiply on sandy land or dunes. It is not only one of the herbaceous species with rich nutrient but also a pioneer species for sand fixation (Editorial Board of Forage flora of China 1987). The analysis of complete chloroplast (cp) genomes has a broad prospect in understanding plant genetic diversity, plant distribution, and phylogenetic evolution (Yang et al. 2019). In this study, we employed Illumina Hiseq to determine and assemble the complete cp genome of *P. flaccidum*, to provide novelty and information on the understanding of its phylogenetic status.

The fresh leaves of *P. flaccidum* were collected from Southwestern Mu Us Sandy Land, China (106°24′E, 37°57′N) and dried with silica gel. A specimen (R02) was deposited in the Herbarium of School of Agriculture, Ningxia University (https://nxy.nxu.edu.cn/, Wangsuo Liu liuwangsuo@sina.com) under the voucher number 6401812020-LW001. The modified CTAB method was used to extract the whole-genomic DNA (Stefanova et al. 2013). The Illumina Hiseq PE150 sequencing was conducted by Biomarker Technologies Co., Ltd. (Qingdao, China). After removal and filtration of low-quality reads, in total, 5 Gb clean reads (the average Q30 was 92.14%) were obtained. The qualified reads were assembled using SPAdes version 3.11.0 software 

**Table:** 




Q5 (Bankevich et al. 2012). The genome annotation was performed by GeSeq (Tillich et al. 2017). Draw annotation map was used by OGDRAW (Greiner et al. 2019) in web service (https://chlorobox.mpimp-golm.mpg.de/OGDraw.html). The complete cp genome 

**Table:** 




Q3 sequence and annotation of *P. flaccidum* were submitted to GenBank (accession No. MW442087).

The borders of IR, LSC, and SSC regions were identified using an online tool, IRscope (Amiryousefi et al. 2018). The cp genome of *P. flaccidum* was 138,336 bp in length and with four typical regions, containing two inverted repeats (IRa and IRb: 22,293 bp), a large single copy (LSC: 81,329 bp), and a small single copy (SSC: 12,421 bp) region. A total of 131 genes were annotated, including seven *rRNA* genes, 38 *tRNA* genes, and 86 protein-coding genes. The GC content of the cp genome was 38.63%. In these genes, there were 93 unique genes and 19 were duplicated in the IR regions. The 19 duplicates including eight protein-coding genes (*rps19*, *rpl2*, *rpl23*, *ycf2*, *ndhB*, *rps7*, *rps12*, and *rps15*), eight *tRNA* genes (*trnH-GUG*, *trnl-CAU*, *trnL-CAA*, *trnV-GAC*, *trnl-GAU*, *trnA-UGC*, *trnR-ACG*, and *trnN-GUU*), and three *rRNA* genes (*rrn4.5*, *rrn5*, and *rrn16*).

In order to clarify the phylogenetic location of *P. flaccidum*, the cp genome sequence of 12 Poaceae was downloaded from NCBI and added three whole cp genome sequences of *Pennisetum* (SY001901, SY001902, and SY001903) at https://lcgdb.wordpress.com/. All cp genomes were aligned by MAFFT version 7.037 (Katoh and Standley 2013). Maximum-likelihood (ML) tree was constructed by PhyML-3.1(GTR) (Guindon et al. 2010) with 1000 bootstrap replications, and the diagram was processed by FigTree version 1.4.4 as shown in the Figure 1. The result showed that *P. flaccidum* closely clustered with *P. cetaceum*. Our study provides a reference for the phylogenetic and taxonomic status of *Pennisetum* in Poaceae.

**Figure 1.**
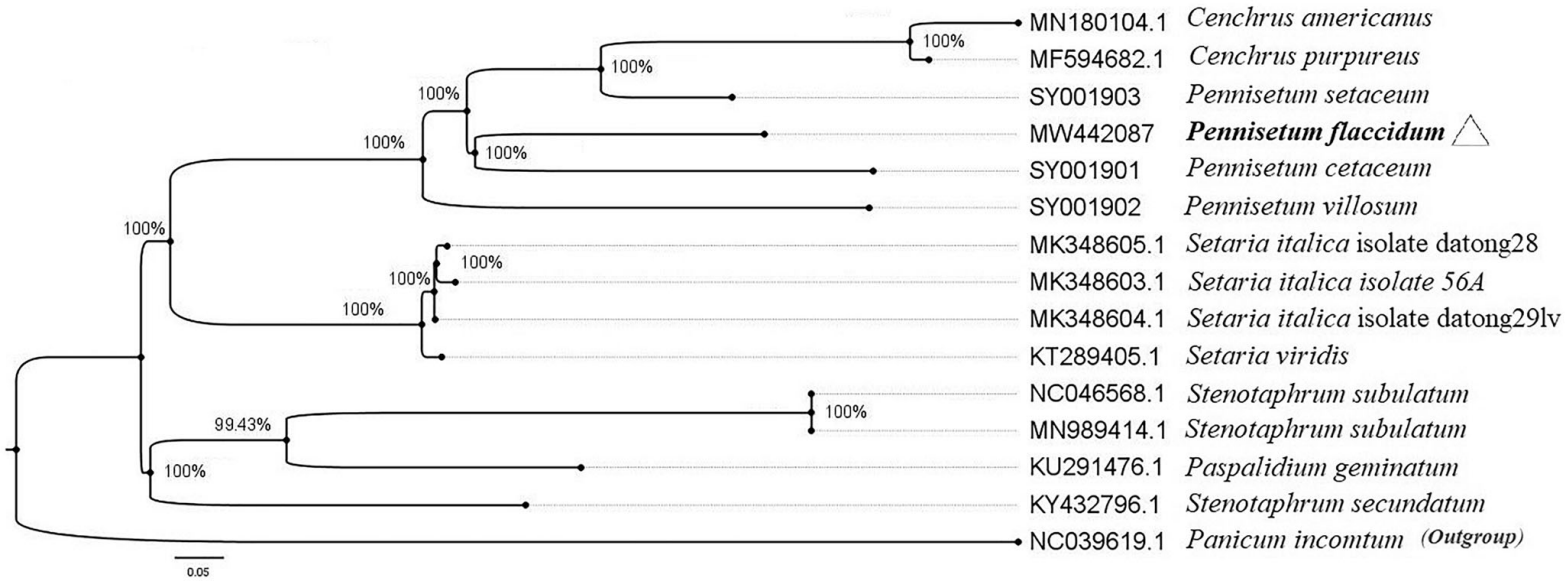
Maximum-likelihood phylogenetic tree based on 14 complete cp genomes. The plant species targeted by a hollow triangle is *Pennisetum flaccidum* in our study.

## Data Availability

The data that support the findings of this study are openly available in NCBI database at https://www.ncbi.nlm.nih.gov/, reference number MW442087. The associated BioProject, Biosample, and SRA numbers are PRJNA689090, SAMN17199738, and SRS7976986, respectively.
